# The Relationship between the Intrapartum Experience and the Risk of Postpartum Depression among Jordanian Women: A Cross-Sectional Study

**DOI:** 10.34763/jmotherandchild.20242801.d-24-00036

**Published:** 2024-12-15

**Authors:** Ayah Fraihat, Lina Abdelfattah, Leen Hajeer, Deema Noaman, Aya Alfaleh, Fida Thekrallah

**Affiliations:** School of Medicine, The University of Jordan, Amman 11942, Jordan; Department of Obstetrics and Gynecology, School of Medicine, The University of Jordan, Amman 11942, Jordan

**Keywords:** Childbirth, Edinburgh postnatal depression scale, postpartum depression

## Abstract

**Background:**

This study seeks to investigate the relationship between birth experience and risk of developing postpartum depression among Jordanian women. Furthermore, it aims to explore the prevalence and risk factors of postpartum depression and raise awareness of it among Jordanians.

**Material and methods:**

This study was carried out on 214 postpartum Jordanian women at Jordan University Hospital (JUH). A questionnaire was handed to participants which touched on demographics, intrapartum experience, along with the Edinburgh Postnatal Depression Scale (EPDS), and the psychosocial factors affecting them.

**Results:**

Among the 214 respondents, 184 women (86%) had postpartum depression according to EPDS, those with high scores and statistically significant p-values include individuals diagnosed with depression (15.89, p = 0.163), those who had previous consultations with a mental health specialist (16.61, p = 0.037), individuals under 18 (18.75, p = 0.028), those who underwent vaginal delivery (15.12, p = 0.008), underwent episiotomy (15.92, p = 0.023), lacked support from the medical team (13.21, p < 0.001), expressed dissatisfaction with childbirth care (17.03, p < 0.001), experienced body image issues during pregnancy (15.74, p = 0.008) and after birth (16.11, p = 0.001), felt anxiety about motherhood (15.88, p < 0.001), reported insufficient emotional support during pregnancy (17.49, p < 0.001), and after childbirth (17.00, p < 0.001).

**Conclusion:**

A significant proportion of Jordanian women are at an increased risk of developing postpartum depression. A maternal age under 18, normal vaginal delivery including episiotomy, and lack of support among others were identified as risk factors for postpartum depression (PPD).

## Introduction

The intrapartum period is an extremely critical part of the entire pregnancy period. It is a life-changing event for any woman, as it could be worrisome to the mother and could be met with feelings of vulnerability and fear.

The birthing experience, which includes any event occurring from the first stage of labour until the end of delivery, can vary widely between women based on different factors such as the presence of emotional support, feeling of self-control and autonomy during labour, perceived levels of pain, physical complications, and undergoing unexpected medical interventions.

Moreover, the satisfaction with the birthing experience differs across institutions, influenced by the quality of care, healthcare professionals’ communication skills, and the labour room environment.

A previous study in Jordan [1] showed that 75.6% of women giving birth using public sector health services were dissatisfied with the intrapartum care. While another study [2] reported that responding to women’s physical and psychological needs during labour and applying less interventions could improve women’s birth satisfaction.

This intrapartum experience can impact the risk of postpartum depression (PPD), a prevalent condition affecting many women during the postpartum period. Yakupova et al. [3] showed that satisfaction with childbirth was significantly inversely associated with PPD.

PPD is more prevalent in the Middle East than in other parts of the world [4]. Unfortunately, cases often go unnoticed due to a lack of awareness, reporting, and screening. The repercussions of PPD on both the mother and child can be detrimental. It could affect child development [5], and in its extreme forms, could lead to maternal thoughts of self-harm and suicidal ideation [6]. Studying the link between the intrapartum experience and PPD has been the concern of many researchers worldwide. In Jordan, more studies are needed to identify the consequences of childbirth on women’s psychological well-being [7]. This study aims to fill this literature gap by being the first of its kind in Jordan to cover both the intrapartum experience of women in labour and PPD, as well as shed the light on this population that has received limited attention in research.

The primary objective of the study is to investigate the relationship between the intrapartum experiences of Jordanian women and their risk of developing PPD. Additionally, it aims to explore the prevalence of PPD in Jordanian women, study the risk factors associated with this condition, and raise awareness of PPD amongst the Jordanian population.

## Material and methods

### Research aim and question

This study aimed to identify the potential relationship between the intrapartum experience and the risk of developing PPD in Jordanian women of childbearing age, considering how debilitating PPD is, combined with the scarcity of research on the influence of the birth experience on the risk of developing PPD, our research question was defined as:

*“What is the potential association between the intrapartum experience and postpartum depression in Jordanian women?”*

### Study design

A descriptive cross-sectional retrospective study design was used. This study was conducted at Jordan University Hospital (JUH), a tertiary referral hospital in Jordan, and was approved by its Institutional Review Board.

### Sampling and data collection

This study focused on women in Jordan. All women who gave birth in Jordan between May 2023 and January 2024 were eligible. The sample included 214 postpartum women selected randomly from the JUH paediatrics clinic.

Data collection was carried out between November 2023 and January 2024. Every day, women who presented to the paediatric clinic at JUH with a baby aged between 2 weeks to 6 months were included in the study until a suitable sample was reached. Data were collected from these postpartum women via an e-questionnaire in a face-to-face setting. A time period of at least 2 weeks between delivery and filling out the questionnaire was required.

The questionnaire was composed of four sections; the first section included basic demographic questions such as age, academic level, occupation, monthly income, and parity. The second section was concerned with the current intrapartum experience and mainly included questions about the mode of delivery (vaginal/c-section), pain during labour, effectiveness of pain relief techniques, episiotomy, complications of delivery, satisfaction with overall intrapartum care, having the opportunity to make decisions as well as being informed of decisions made in the delivery room, opportunities to talk to a healthcare professional about their feelings in relation to the birth, and the physical conditions of the birth environment.

The third section was the Edinburgh Postnatal Depression Scale (EPDS) which is a set of ten screening questions used by health professionals to detect mothers at risk of PPD. These ten questions investigate how the mothers have been feeling the previous seven days, including feeling sad, having difficulty sleeping, feeling anxious or worried for no good reason, thinking of self-harm, etc. Each item has a score of 0–3 according to the increased severity of the symptom, a scoring system with cut-off points (7–13) to identify mild depression, (14–19) for moderate depression, and (19–30) for severe depression was applied. A systematic review and meta-analysis [8] compared the predictive validity of EPDS and other tools for screening depression in pregnant and postpartum women showed that EPDS can be used in preference to other screening tools.

The fourth and final section explored the psychosocial aspect of the patients’ life and took into consideration the psychiatric history of the patient, illicit drug use, and alcohol consumption as well as questions about the patient’s support network, financial challenges, body image issues, medical complications during pregnancy, and their effect on the baby. A comparative study [9] revealed that social support is a significant, protective factor for PPD, and the variety of support providers in a woman’s social network is important, especially in the context of family type.

The questionnaire was validated by 30 patients and then edited accordingly before the official data collection began.

### Statistical analysis

The data utilised in this study were initially collected through Google Forms and subsequently transformed into an Excel spreadsheet format prior to integration into the Statistical Package for Social Sciences (SPSS) version 26. To comprehensively analyse the dataset, a descriptive analysis approach was employed, portraying categorical variables in terms of percentages and frequencies, while numerical variables were represented using the mean and standard deviation (SD) to provide a quantitative assessment of the data. The normality of the data distribution was examined through the Shapiro–Wilk test.

Furthermore, potential differences in means among variables were explored utilising the Mann–Whitney U test and Kruskal–Wallis tests. A significance threshold of p < 0.05 was adopted, deeming results with a p-value below this threshold as statistically significant.

## Results

### Demographic and maternal pregnancy experience

A total of 214 respondents completed the questionnaire. In terms of age distribution, over half fell within the 25–35 years age group, comprising 111 individuals (51.9%). Ninety-nine respondents held a bachelor’s degree as their highest level of education. Monthly income between 400 and 1000 JDs was reported by 120 participants (56.1%). Regarding employment status, only 79 respondents were employed (36.9%).

One hundred and twenty-five participants reported having two or more pregnancy experiences, constituting 58.4% of the sample. Concerning the place of birth, the majority, 167 respondents (78.0%), reported delivering at JUH. Vaginal delivery was the choice for more than half of the participants, with 111 individuals (51.9%) opting for this method. Among those who had a vaginal delivery, 71 reported the need for an episiotomy, accounting for 64.0% of this subgroup.

**Figure 1.**
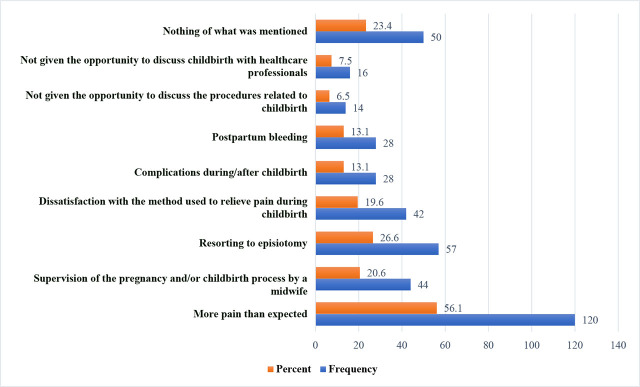
The experience of pregnancy and childbirth.

A total of 34 participants (15.9%) indicated that they brought companions into the delivery room. Additionally, 162 respondents (75.7%) reported receiving support from doctors and all medical staff, while 152 expressed satisfactions with the provided care (71.0%). Regarding the experience of pregnancy and childbirth, 120 reported that the pain was out of control (56.1%). Figure 1 shows the estimate of maternal experience through pregnancy and childbirth. When asked about their room experience and comfort assessment, 96 reported that the room was spacious and suitable (44.9%) while 93 stated that the room was adequately lit (43.5%).

**Figure 2.**
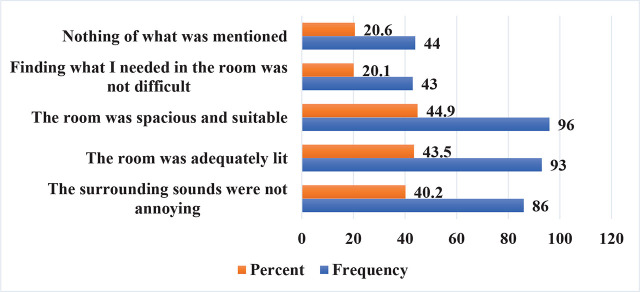
Room experience and comfort assessment.

**Figure 3.**
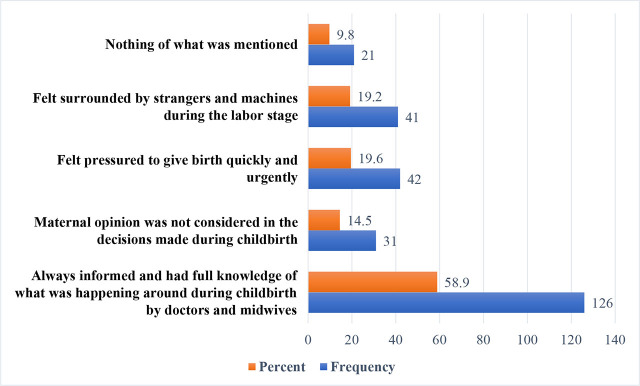
Decision making and pressures in the delivery room.

Figure 2 summarised the estimate of maternal room experience. Furthermore, when asked about their experience in the delivery room, 120 mothers reported that they were always informed and had full knowledge of what was happening around during childbirth by doctors and midwives (56.1%), and Figure 3 shows the estimate of maternal decision making and pressures in the delivery room.

Financial challenges were acknowledged by 79 participants (36.9%) who reported facing a recent financial crisis. Furthermore, 121 respondents (56.5%) stated that their pregnancy was planned, and 172 participants (80.4%) conveyed that it was desired. Health complications during pregnancy were reported by 71 individuals (33.2%). Among this group, 39 participants (18.2%) mentioned that these complications had an impact on their infants.

Moreover, 78 respondents (36.4%) reported facing problems related to body image during pregnancy, and 81 participants (37.9%) stated ongoing challenges related to body image after childbirth. One hundred and twenty-seven individuals (59.3%) mentioned that thinking about the motherhood stage causes feelings of anxiety or doubt. Finally, six mothers (2.8%) disclosed the use of any type of addictive substances. Table 1 demonstrates the frequencies of the participants’ demographic and their pregnancy experience.

**Table 1.** Demographic and maternal pregnancy experience. (n = 214)

**Item**	**Frequency (%)**
**Maternal age**	
Less than 18 years	4 (1.9)
18–25 years	62 (29.0)
25–35 years	111 (51.9)
More than 35 years	37 (17.2)
**Education level**	
Primary	13 (6.1)
High school	57 (26.6)
Diploma	28 (13.1)
Bachelor	99 (46.3)
Higher grades	17 (7.9)
**Family monthly income**	
Less than 400 JDs	58 (27.1)
400 – 1000 JDs	120 (56.1)
More than 1000 JDs	36 (16.8)
**Job status**	
Employed	79 (36.9)
Not employed	135 (63.1)
**Current pregnancy status**	
First pregnancy	89 (41.6)
Second or more	125 (58.4)
**Place of birth**	
Jordan University Hospital (JUH)	167 (78.0)
Other hospitals	47 (22.0)
**Type of current pregnancy**	
Vaginal delivery	111 (51.9)
Caesarean section	103 (48.1)
**There was a need for an episiotomy during childbirth for those who done vaginal delivery**	
Yes	71 (64.0)
No	40 (36.0)
**Bringing a companion into the delivery room was allowed**	
Yes	34 (15.9)
No	176 (82.2)
Did not ask	4 (1.9)
**Encouragement and support were received from the supervising doctor, midwives, and medical staff**	
Yes	162 (75.7)
No	52 (24.3)
**Satisfaction was felt with the care provided during the childbirth process**	
Yes	152 (71.0)
No	62 (29.0)
**The financial crisis had been experienced recently**	
Yes	79 (36.9)
No	135 (63.1)
**The pregnancy was planned**	
Yes	121 (56.5)
No	93 (43.5)
**The pregnancy was desired**	
Yes	172 (80.4)
No	42 (19.6)
**Health complications were experienced during pregnancy**	
Yes	71 (33.2)
No	143 (66.8)
**Among individuals who disclosed experiencing health complications during pregnancy, there is a consideration of the potential impact of these health issues on the infant’s well-being**	
Yes	25 (35.2)
No	46 (64.8)
**The child suffers from health problems (such as congenital deformities or genetic diseases)**	
Yes	39 (18.2)
No	175 (81.8)
**Problems related to body image during pregnancy were experienced**	
Yes	78 (36.4)
No	136 (63.6)
**Currently experiencing problems related to body image after childbirth**	
Yes	81 (37.9)
No	133 (62.1)
**Thinking about the motherhood stage caused feelings of anxiety or doubt**	
Yes	127 (59.3)
No	87 (40.7)
**The use of any type of addictive substances (drugs, alcohol, or others) was acknowledged**	
Yes	6 (2.8)
No	208 (97.2)

JDs: Jordanian dinar.

### Current mental health status and background

Nineteen respondents (8.9%) disclosed having received a diagnosis of depression from a specialist doctor, while 23 individuals (10.7%) reported previous consultations with a mental health specialist, primarily for depression in 15 cases (65.2%). Twelve respondents (5.6%) acknowledged a positive family history, with obsessive compulsive disorder (OCD) being the most frequently reported condition among them (33.3%). Half of the respondents (50.5%) indicated experiencing recent situations that heightened their stress levels.

Furthermore, 74 respondents (34.6%) reported encountering recent challenges in their relationships with spouses or family members. Additionally, 49 participants (22.9%) expressed a lack of emotional support from family and friends during pregnancy.

Finally, 57 individuals (26.6%) reported feelings of inadequate psychological support from family and friends after childbirth. Table 2 demonstrates the frequencies of current mental health status and background.

**Table 2.** Current mental health status and background. (n = 214)

**Item**	**Frequency (%)**
**Diagnosis of depression by a specialist doctor has been experienced**	
Yes	19 (8.9)
No	195 (91.1)
**Consultation with a specialist in the field of mental health has taken place**	
Yes	23 (10.7)
No	191 (89.3)
**The reason for the consultation is explored among those who consulted specialist before**	
Depression	15 (65.2)
Anxiety	3 (13.0)
Seeking help	2 (8.7)
No specific reasons	3 (13.0)
**There is someone in the family suffering from mental illnesses**	
Yes	12 (5.6)
No	202 (94.4)
**The specific illness is identified in the family among those who had positive family history**	
OCD	4 (33.3)
Bipolar	3 (25.0)
PTSD	1 (8.3)
Depression	2 (16.7)
Can not recall	2 (16.7)
**Recent situations have been experienced that increased stress**	
Yes	108 (50.5)
No	106 (49.5)
**Recent problems have been faced in the relationship with a spouse or family members**	
Yes	74 (34.6)
No	140 (65.4)
**Lack of emotional support from family and friends during pregnancy was felt**	
Yes	49 (22.9)
No	165 (77.1)
**There is a sense of insufficient psychological support from family and friends after childbirth**	
Yes	57 (26.6)
No	157 (73.4)

OCD: Obsessive-compulsive disorder, PTSD: Post-traumatic stress disorder.

### Edinburgh postnatal depression scale (EPDS)

**Figure 4.**
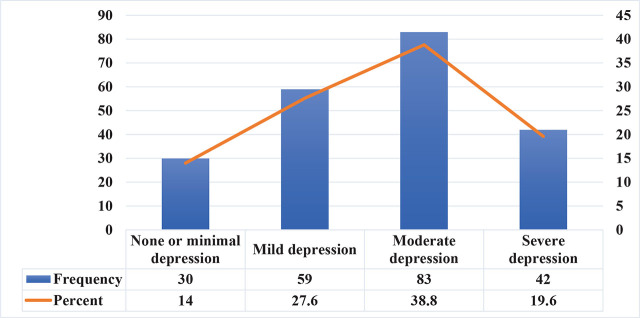
The frequency of EPDS severity.

The mean score was 14.15 ± 6.36 (± SD) with a range of 29 (0–29). Regarding the severity ranges for the EPDS, moderate depression was the most reported 83 (38.8%). Figure 4 demonstrates the frequency of EPDS severity.

### Association between the EPDS score and maternal demographics, pregnancy experiences, and mental health status

The study employed Kruskal–Wallis and Mann–Whitney U tests to explore potential variations in the mean scores of EPDS concerning maternal demographics, pregnancy experiences, and mental health status. Maternal participants aged less than 18 years, as well as those who underwent vaginal delivery, required episiotomy during childbirth, lacked encouragement and support from the supervising medical team, expressed dissatisfaction with provided care during childbirth, encountered body image issues during pregnancy or after childbirth, experienced anxiety about the motherhood stage, sought consultation with a mental health specialist, faced recent stress-inducing situations or problems in relationships with a spouse or family members, reported insufficient emotional support from family and friends during pregnancy, and perceived inadequate psychological support from family and friends after childbirth, exhibited significantly higher mean EPDS scores (p-values = 0.028, 0.008, 0.023, < 0.001, < 0.001, 0.008, 0.001, < 0.001, 0.037, < 0.001, < 0.001, < 0.001, and < 0.001, respectively).

Table 3 demonstrates the association between EPDS mean scores among the included sample.

**Table 3.** Association between the EPDS score and maternal demographics, pregnancy experiences, and mental health status

**Item**	**Mean ± SD**	**P – value**
**Maternal age^A^**		**0.028**
Less than 18 years	18.75 ± 2.22	
18–25 years	15.23 ± 6.05	
25–35 years	14.09 ± 6.28	
More than 35 years	12.05 ± 6.91	
**Education level^A^**		0.463
Primary	15.85 ± 5.60	
High school	13.68 ± 6.33	
Diploma	15.64 ± 6.73	
Bachelor	13.91 ± 6.41	
Higher grades	13.41 ± 6.25	
**Family monthly income^A^**		0.538
Less than 400 JDs	14.29 ± 7.07	
400 – 1000 JDs	14.36 ± 5.91	
More than 1000 JDs	13.25 ± 6.69	
**Job status^B^**		0.372
Employed	14.61 ± 6.42	
Not employed	13.89 ± 6.34	
**Current pregnancy status^B^**		0.132
First pregnancy	14.78 ± 6.22	
Second or more	13.71 ± 6.45	
**Place of birth^B^**		0.435
Jordan University Hospital	14.01 ± 6.49	
Other hospitals	14.66 ± 5.92	
**Type of current pregnancy^B^**		0.008
Vaginal delivery	15.12 ± 6.20	
Caesarean section	13.12 ± 6.40	
**There was a need for an episiotomy during childbirth for those who did vaginal delivery^B^**		0.023
Yes	15.92 ± 6.67	
No	13.70 ± 5.04	
**Bringing a companion into the delivery room was allowed^B^**		0.352
Yes	14.85 ± 6.07	
No	13.90 ± 6.42	
**Encouragement and support were received from the supervising doctor, midwives, and medical staff^B^**		<0.001
Yes	13.21 ± 6.30	
No	17.10 ± 5.67	
**Satisfaction was felt with the care provided during the childbirth process^B^**		< 0.001
Yes	12.98 ± 6.29	
No	17.03 ± 5.61	
**The financial crisis was experienced recently^B^**		< 0.001
Yes	16.08 ± 5.84	
No	13.03 ± 6.41	
**The pregnancy was planned^B^**		0.140
Yes	13.55 ±6.53	
No	14.94 ± 6.08	
**The pregnancy was desired^B^**		0.101
Yes	13.83 ± 6.47	
No	15.48 ± 5.79	
**Health complications were experienced during pregnancy^B^**		0.054
Yes	15.38 ± 5.79	
No	13.55 ± 6.56	
**Among individuals who disclosed experiencing health complications during pregnancy, there is a consideration of the potential impact of these health issues on the infant’s well-being^B^**		0.382
Yes	16.32 ± 4.77	
No	14.87 ± 6.27	
**The child suffers from health problems (such as congenital deformities or genetic diseases)^B^**		0.134
Yes	15.72 ± 5.34	
No	13.81 ± 6.53	
**Problems related to body image during pregnancy were experienced^B^**		0.008
Yes	15.74 ± 6.23	
No	13.24 ± 6.28	
**Currently experiencing problems related to body image after childbirth^B^**		0.001
Yes	16.11 ± 6.30	
No	12.96 ± 6.12	
**Thinking about the motherhood stage causes feelings of anxiety or doubt^B^**		< 0.001
Yes	15.88 ± 6.04	
No	11.63 ± 5.99	
**The use of any type of addictive substances (drugs, alcohol, or others) is acknowledged^B^**		0.053
Yes	18.67 ± 3.88	
No	14.02 ± 6.38	
**Diagnosis of depression by a specialist doctor has been experienced^B^**		0.163
Yes	15.89 ± 7.02	
No	13.98 ± 6.29	
**Consultation with a specialist in the field of mental health has taken place^B^**		0.037
Yes	16.61 ± 6.30	
No	13.86 ± 6.32	
**The reason for the consultation is explored among those who consulted specialist before^A^**		0.973
Depression	16.53 ± 7.29	
Anxiety	15.67 ± 6.43	
Seeking help	17.50 ± 4.95	
No specific reasons	17.33 ± 2.89	
**There is someone in the family suffering from mental illnesses^B^**		0.327
Yes	15.83 ± 5.56	
No	14.05 ± 6.41	
**The specific illness is identified in the family among those who had positive family history^A^**		0.760
OCD	15.00 ± 5.42	
Bipolar	16.33 ± 5.69	
PTSD	18.00 ± 0.00	
Depression	19.50 ± 4.95	
**Recent situations have been experienced that increased stress^B^**		< 0.001
Yes	16.19 ± 5.29	
No	12.08 ± 6.71	
**Recent problems have been faced in the relationship with a spouse or family members^B^**		< 0.001
Yes	16.85 ± 5.22	
No	12.73 ± 6.47	
**Lack of emotional support from family and friends during pregnancy was felt^B^**		< 0.001
Yes	17.49 ± 5.97	
No	13.16 ± 6.15	
**There is a sense of insufficient psychological support from family and friends after childbirth^B^**		< 0.001
Yes	17.00 ± 6.53	
No	13.12 ± 6.00	

JDs: Jordanian dinar, OCD: Obsessive-compulsive disorder, PTSD: Post-traumatic stress disorder, and SD: standard deviation.

A: Kruskal–Wallis test,

B: Mann–Whitney U test.

## Discussion

A total of 214 Jordanian women participated in this study. Of those, 184 women (86%) had PPD according to the EPDS score. These are classified according to severity as follows: 59 participants (27.6%) had mild depression, 83 participants (38.8%) had moderate depression, and 42 participants (19.6%) had severe depression. A previous descriptive study carried out in Jordan [10] concluded that the prevalence of PPD among Jordanian women is 52.8%, with a cut off score of ≥12 indicating probable depression. In our study, we adopted a scoring system with cut-off points (7–13) to identify mild depression, (14–19) for moderate depression, and (19–30) for severe depression, aiming to enhance the test’s sensitivity.

One notable demographic factor that emerged as a significant contributor to elevated EPDS scores was maternal age. Participants aged less than 18 years exhibited significantly higher mean EPDS scores compared to those older than 18. This is concordant with the results of [11]’s study, which found decreasing risks of antenatal depressive symptoms with increasing maternal age, with the youngest mothers (<23 years) at two or more times the risk of depressive symptoms compared with older mothers. This suggests a potential vulnerability among adolescent mothers in terms of postnatal depressive symptoms, highlighting the importance of targeted interventions and support for this demographic group.

Other factors which were found to affect EPDS score are related to the mode of delivery and the birth process. Participants who underwent vaginal delivery reported higher mean EPDS scores. This is discordant with [12] which revealed that compared to vaginal delivery, women who underwent caesarean delivery tended to have higher risk of PPD. Another study [13] found that delivering by spontaneous vaginal birth, elective caesarean section, or emergency caesarean section had no effect on EPDS scores.

Another factor was the use of episiotomy in those who underwent vaginal delivery. Participants who required episiotomy during childbirth reported higher mean EPDS scores. This is supported by the study [14] which found that the total EPDS score was significantly higher among mothers with episiotomy than those with intact perineum. This can be explained by the traumatic birth experience, the pain and discomfort associated with episiotomy in the postdelivery period, decreased mobility, and a more extended and challenging physical recovery.

Furthermore, the study identified the impact of the level of care provided during childbirth on the mental health of the mother. Maternal participants who lacked encouragement and support from the supervising medical team and expressed dissatisfaction with provided care during childbirth exhibited significantly higher mean EPDS scores. This is supported and explained by Khalat et al.’s [15] interventional study in which the intervention group was provided care based on WHO recommendations on RMC and EC; the control group received routine care. It concluded that implementing respectful maternity care and effective communication during labour and delivery can reduce the duration of labour and hospitalisation, decrease emergency caesarean deliveries and labour pain, and promote neonatal outcomes. Another study [3] revealed that perceived severity of labour and worse well-being after delivery were associated with lower birth satisfaction. On the other hand, the presence of a partner and a personal midwife or doula at birth was associated with higher birth satisfaction. This indicates the importance of the individual perceived care and support during labour as a possible avenue for PPD prevention.

Additionally, the study identified the impact of social support and interpersonal relationships in mitigating postnatal depressive symptoms. Maternal participants who reported insufficient emotional support from family and friends during pregnancy, and perceived inadequate psychological support from family and friends after childbirth exhibited higher mean EPDS scores. This is concordant with Dibaba et al.’s [16] study, which showed that women with high social support were 0.26 times as likely as women with low or no social support to experience PPD. This highlights the critical role of a supportive social environment in mitigating the adverse effects of perinatal stressors and reducing the tendency to developing PPD.

Not only external environmental factors were found to impact the development of PPD, but also individual psychological factors were found to impact the EPDS scores. Maternal participants who experienced body image issues during pregnancy or after childbirth, encountered anxiety about the motherhood stage, sought consultation with a mental health specialist, faced recent stress-inducing situations, or encountered problems in relationships with a spouse or family members exhibited significantly higher mean EPDS scores. This is supported by the results of Çankaya’s [17] study which found that stress levels perceived by the women with PPD during pregnancy were significantly higher than those without PPD according to the Perceived Stress Scale (PSS). Anxiety (HADS-A, scores above 10) and depression (HADS-D, scores above 7) were found to be significantly higher in women with PPD compared to those without PPD. This supports the importance of considering individual mental health and well-being as a distinct factor that influences the development of PPD.

Getting pregnant at a young age, especially under 18, is linked to a higher chance of PPD. Knowing about pregnancy early on and its impact for young married women can really help prevent PPD. It is important to have a well-trained team during childbirth, including an obstetrician, nurses who take care of both nursing and keeping the patient informed about labour, and an anaesthesiologist for pain relief. It is also a good idea for pregnant women to talk to a psychologist during and after pregnancy to avoid any psychological issues.

The limitations of this study include the small sample size that was only conducted from JUH. Therefore, the results cannot be generalised to all Jordanian women. Also, the retrospective approach increases the susceptibility for memory bias.

For future research projects based on our study, we recommend collecting data from different regions in the country where the study is happening. This way, we can get more generalised and reliable results. We also encourage using a prospective study design where the women are interviewed directly after delivery and followed up to assess PPD.

## Conclusion

In conclusion, due to the high risk of developing PPD among Jordanian mothers and due to the strong association between the intrapartum experience and the development of PPD, immediate attention and action from policy makers at the national level becomes critical. Several modifications on the antenatal and intrapartum care are required, that may prevent the harmful consequences of PPD on the mother, newborn and their family.

Prioritising mental health as a public health priority and allocate sufficient resources to support evidence-based interventions aimed at reducing the prevalence of PPD are required, this may include increasing funding for mental health services,

Regular campaigns targeting both women in their reproductive age and caregivers are essential to address the widespread prevalence of this issue. Additionally, training caregivers on effective strategies to manage this problem is crucial.

Before delivery every woman who is at risk of developing PPD should be identified to increase their awareness about PPD by teaching them and their partners and family members about the signs and symptoms of PPD which can help in early detection and management [18].

In the antenatal period, screening for depressive symptoms can be done to identify women at an increased risk of PPD. A meeting between the mother and the gynaecologist before delivery to clarify the mother’s expectations regarding the intrapartum experience to work towards meeting them may help alleviate the dissatisfaction of the intrapartum care. As part of counselling, caregivers should discuss potential complications that might occur during labour, the unexpected need for procedures like episiotomy and instrumental delivery, and their indications with patients.

Furthermore, caregivers should provide support to the patient, engage in shared decision-making processes, obtain consent before each step, ensure a comfortable and spacious room, prioritise the room setting as it significantly impacts the experience, and allow the spouse to join to provide support during labour.

### Abbreviations

PPD: Postpartum Depression
EPDS: Edinburgh Postnatal Depression Scale
SD: Standard Deviation
OCD: Obsessive Compulsive Disorder
PSS: Perceived Stress Scale
PTSD: Post-traumatic stress disorder.
JDs: Jordanian dinars
SPSS: Statistical Package for Social Sciences
JUH: Jordan University Hospital

### Availability of data and materials

The datasets used and/or analysed during the current study are available from the corresponding author upon reasonable request.

### Ethics approval and consent to participate

The Institutional Review Board at Jordan University Hospital approved the study protocol. Written informed consent was obtained from all participants.

### Consent for publication

Written consent was obtained from all participants.

### Key points

- This study aims to find the potential association between birth experience and the development of postpartum depression (PPD), by exploring its incidence and risk factors.
- A questionnaire covering demographics, intrapartum experience and EPDS score for PPD was used among 214 postpartum Jordanian women using a descriptive cross-sectional retrospective design.
- A significant proportion of women with PPD were associated with factors such as younger age, vaginal delivery and episiotomy, lack of medical team support, dissatisfaction with childbirth care, and insufficient psychological support during and after pregnancy.
- It’s crucial to prioritise postpartum mental health as a public health priority and allocate sufficient resources to support interventions aimed at reducing the prevalence of PPD, starting with increasing awareness, optimising medical care and support, and implementing screening programs.
